# Deficiency of TRIM32 Impairs Motor Function and Purkinje Cells in Mid-Aged Mice

**DOI:** 10.3389/fnagi.2021.697494

**Published:** 2021-08-06

**Authors:** Jian-Wei Zhu, Wei-Qiang Jia, Hui Zhou, Yi-Fei Li, Ming-Ming Zou, Zhao-Tao Wang, Bing-Shan Wu, Ru-Xiang Xu

**Affiliations:** ^1^Department of Neurosurgery, Sichuan Provincial People's Hospital, University of Electronic Science and Technology of China, Chengdu, China; ^2^Department of Pediatrics, Chengdu Children Special Hospital, Chengdu, China; ^3^Department of Neurosurgery, Peking University Shenzhen Hospital, Shenzhen, China; ^4^Department of Neurosurgery, The Seventh Medical Center of PLA General Hospital, Beijing, China; ^5^Department of Neurosurgery, The Second Affiliated Hospital of Guangzhou Medical University, Guangzhou, China; ^6^Department of Neurosurgery, The First Affiliated Hospital of Anhui Medical University, Hefei, China

**Keywords:** cerebellum, TRIM32, motor coordination (MC), purkine cell, cerebellar degeneration, INPP5A

## Abstract

Proper functioning of the cerebellum is crucial to motor balance and coordination in adult mammals. Purkinje cells (PCs), the sole output neurons of the cerebellar cortex, play essential roles in cerebellar motor function. Tripartite motif-containing protein 32 (TRIM32) is an E3 ubiquitin ligase that is involved in balance activities of neurogenesis in the subventricular zone of the mammalian brain and in the development of many nervous system diseases, such as Alzheimer's disease, autism spectrum disorder, attention deficit hyperactivity disorder. However, the role of TRIM32 in cerebellar motor function has never been examined. In this study we found that motor balance and coordination of mid-aged TRIM32 deficient mice were poorer than those of wild-type littermates. Immunohistochemical staining was performed to assess cerebella morphology and TRIM32 expression in PCs. Golgi staining showed that the extent of dendritic arborization and dendritic spine density of PCs were decreased in the absence of TRIM32. The loss of TRIM32 was also associated with a decrease in the number of synapses between parallel fibers and PCs, and in synapses between climbing fibers and PCs. In addition, deficiency of TRIM32 decreased Type I inositol 1,4,5-trisphosphate 5-phosphatase (INPP5A) levels in cerebellum. Overall, this study is the first to elucidate a role of TRIM32 in cerebellar motor function and a possible mechanism, thereby highlighting the importance of TRIM32 in the cerebellum.

## Introduction

In mammals, the cerebellum is an important component of the motor pathways of the central nervous system (CNS) and plays an essential role in motor coordination (Mauk et al., 2000; Glickstein et al., 2009). The specific organizational structure and characteristic neuronal circuitry of the cerebellum is crucial for the proper functioning of motor coordination and motor learning. Histologically, the adult cerebellar cortex is a trilaminar structure comprised of, from inside to outside, an internal granule cell layer (GCL), a monolayer of Purkinje cells containing the somata of Purkinje cells (PCs) and Bergmann glia and a molecular layer containing interneurons, the dendrites of PCs, radial fibers of Bergmann glia, and axons of granule cells (Wang and Zoghbi, 2001). PCs, the sole output neurons of the cerebellar cortex, are indispensable for activation of the cerebellar neuronal circuitry (Saywell et al., 2014). The primary dendrites of PCs extend toward molecular layer and branch extensively to form secondary and tertiary dendrites, which ultimately form elaborate dendritic arbors with characteristic and recognizable branching patterns. In the adult cerebellum, PCs form synapses with two afferent input fibers in order to convey information: (i) the parallel fibers (PFs), the axons of granule cells, connect with PCs through the spines located on the distal part of the dendritic tree to form synapses between the PFs and PCs (PFs-PCs), and (ii) extensive synaptic contacts between climbing fibers (CFs) originating from the inferior olive nuclei and the proximal dendritic shaft of PCs that form synapses between CFs and PCs (CFs-PCs) (Hansel et al., 2001; Ito, 2002; Tanaka, 2009). Thus, dendritic arborization and the dendritic spine density of PCs, as well as the numbers of PFs-PCs and CFs-PCs, underlie crucial aspects of cerebellar function.

Cerebellar degeneration is closely associated with cerebellar motor dysfunction or ataxia (Yang et al., 2018; Liu et al., 2020). The progressive and permanent loss of PCs is a hallmark of many ataxias (Yang et al., 2015). Type I inositol 1,4,5-trisphosphate 5-phosphatase (INPP5A), a terminator of the inositol 1,4,5-triphosphate (IP_3_) second messenger, is abundant in the cerebellum, especially in PCs (Berridge, 2016; Liu et al., 2020). Deletion of INPP5A leads to PCs degeneration and ataxia in mice and contributes to cerebellar degeneration in Spinocerebellar ataxia type 17 (SCA17) mice, whereas overexpression of INPP5A in the cerebellum ameliorated PCs degeneration in SCA17 knock-in mice (Yang et al., 2015; Liu et al., 2020). Therefore, INPP5A is critical to PCs survival and execution of cerebellar motor function.

Tripartite motif containing protein 32 (TRIM32), a member of the TRIM protein family, has been implicated in a number of diverse biological activities in the CNS. As a determinant of cell fate, TRIM32 regulates the differentiation of neural progenitor cells in the embryonic and adult stages of life (Schwamborn et al., 2009; Hillje et al., 2013). Moreover, the loss of TRIM32 leads to inhibition of neuronal differentiation causing accumulation of adult phase olfactory bulb neurons, which ultimately results in impaired olfactory capabilities (Hillje et al., 2015). A recent study by our group revealed that TRIM32 deficiency reduced the populations of GABAergic interneurons in the cortex and hippocampus (Zhu et al., 2019). In addition, TRIM32 participated in many CNS-related diseases, including Alzheimer's disease (Yokota et al., 2006), autism spectrum disorder (ASD) and attention deficit hyperactivity disorder (ADHD) (Lionel et al., 2011, 2014; Zhu et al., 2019), anxiety and obsessive compulsive disorder (Lionel et al., 2014).

Previous studies revealed that TRIM32 correlates with motor dysfunction related diseases through regulating proteins degradation with ubiquitin-proteasome pathway. TRIM32 regulates myofibrillar protein turnover in physiological situation, and TRIM32 mutant disrupts the ability to degrade dysbindin (Locke et al., 2009) and actin (Cohen et al., 2012) by ubiquitination, which ultimately leads to Limb-gridle muscular dystrophy type 2H (LGMD2H), a characteristic with muscular dystrophic changes and motor dysfunction (Shieh et al., 2011). Amyotrophic lateral sclerosis (ALS), a progressive and fatal motor neuron disease, is also correlated with TRIM32.

A recent study revealed that TRIM32 deficiency enhances the incidence of medulloblastoma formation, as TRIM32 suppresses cerebellar development and tumorigenesis by degrading glioma-associated oncogene/sonic hedgehog signaling (Wang et al., 2019). These data indicated that TRIM32 is possible involved in regulating cerebellar function. Despite these findings, the role of TRIM32 in motor function regulating remains unclear. Here, we report for the first time that TRIM32 deficient mid-aged mice exhibits impaired motor balance, coordination, motor learning. Furthermore, poor motor balance and coordination may be due to pathological alternations of cerebellum combined with TRIM32 deficiency. Motor discoordination usually occurs in subjects with ASD and ADHD (Dewey et al., 2007; Green et al., 2009; Van Waelvelde et al., 2010). Previous studies conducted by our group and others (Lionel et al., 2011, 2014; Zhu et al., 2019) reported that TRIM32 is strongly associated with ASD and ADHD, but the mechanism underlie it remains unclear.

## Materials and Methods

### Mice

For all experiments, *TRIM32*^−^^/–^ mice (Mixed 129SvEvBrd × C57BL/6J background) (Zhu et al., 2019) and wild-type (WT) littermate control mice were obtained from heterozygous breeding pairs. All mice were housed under standard laboratory conditions and a 12 h light-dark cycle with ad libitum access to food and water. The protocols of all animal experiments were approved by the Institutional Animal Care and Use Committee of Sichuan Provincial People's Hospital, University of Electronic Science and Technology of China. During the experimental procedures, all efforts were made to minimize the number and suffering of mice.

### Behavioral Analysis

All behavioral tests were performed with the use of younger (6 months old) and mid-aged (10 months old, after 4 months from youngers) *TRIM32*^−/−^ mice (seven males and six females) and WT littermate controls (eight males and seven females, after 4 months from youngers). An experimental paradigm for the behavioral tests is shown in Supplementary Figure 1. All experiments were performed by investigators blind to the genotype of the mice.

### Footprint Test

The forepaws and hindpaws of each mouse were painted with nontoxic red and black ink, respectively. Each mouse was placed at one end of a tunnel (length, 70 cm; width, 10 cm; height, 10 cm) lined with white paper (Zhu et al., 2016) and allowed to walk through into its respective home cage at the other end which was accessible through a hole. Each mouse underwent three pre-trails before performing an additional three trials for data acquisition. For each of six successive paws prints, a point was identified at the base of the middle toe. Stride length was defined as the straight line distance of successive points of contact of the ipsilateral hindpaw. Stride width was defined as the perpendicular line distance of the hindpaw point of contact to that of a line connecting the successive contralateral hindpaw. Inter-limb coordination was defined as the straight line distance between the point of contact of the hindpaw and that of the adjacent ipsilateral forepaw. For measurements, the first and last 10 cm of the prints were excluded. If the mouse stopped in the middle of the track, the trial was repeated. Mice that did not move were excluded.

### Accelerating-Rotarod Test

The accelerating rotarod test was used to evaluate motor coordination, balance, and motor learning, as described previously (Sano et al., 2018), but with slight modifications. Briefly, TRIM32^−/–^ and WT mice were held by the tail and placed on a rotarod at an initial speed of 4 rpm facing away from the direction of rotation. After 10 s, the rod speed was accelerated from 4 to 40 rpm over a period of 4 min and then held at a constant speed of 40 rpm for further 1 min. The time spent on the rotarod before falling was recorded over a maximum observation period of 5 min. Mice were subjected to one trial on day 1 and three trials (inter-trial interval of ≥ 5 min) on days 2–6.

### Ladder Rung Task

The ladder rung test was conducted using an apparatus (Bioseb) composed of two side walls (length, 90 cm; height 20 cm) spaced 5 cm apart to allow for passage, while preventing the mice from turning around. The bottom edge of each wall contained 77 plastic bars (length 7 cm; diameter 0.5 cm). The apparatus was elevated 30 cm above the ground with the respective home cage at the end. Mice were tested under two ladder conditions: a regular pattern, where the rungs were spaced at 1 cm intervals, and an irregular pattern, where the distance between the rungs varied from 1 to 2 cm (Zhu et al., 2016). Each mouse underwent five continuous pre-trials prior to three trials for data acquisition (i.e., crossing time and number of limb errors). For each mouse, the data obtained from three trials were averaged for analysis.

### Hindlimb Clasping Test

Mice were suspended by the tail and the extent of hindlimb clasping was observed for 10 s and scored on a scale of 0–3 as follows: score = 0: both hindlimbs were consistently splayed outward, away from abdomen, with splayed toes; score = 1: only one hindlimb was retracted toward the abdomen or both hindlimbs were partially retracted toward the abdomen, with the splayed toes but not touching the abdomen; score = 2: both hindlimbs were partially retracted toward the abdomen and touching the abdomen, but without touching each other; score = 3: both hindlimbs were entirely clasped and touching the abdomen (Zhu et al., 2016).

### Beam Walk Test

A narrow beam (length, 70 cm; diameter 1 cm) was placed horizontally 60 cm above the platform surface. One end of the beam was fixed to the platform, while the other was attached to a closed bright goal box (25 cm^2^). The tests were conducted after a 2-day training period. Briefly, each mouse was acclimated to the goal box for 3 min and then placed on the beam at a distance of 10 cm away from the goal box. Each mouse was placed at increasing distances of 20, 30, 50, and 70 cm from the goal box and trained to navigate the beam for 3–4 consecutive trails, from each starting point (Zhu et al., 2016). The amount of time for the mouse to cross the full length of the beam to the goal box was recorded. The maximum time allowed to traverse the beam was 120 s. If the mouse fell before reaching at the goal box, the time was recorded as 120 s. The time spent immobilized (frozen) was also recorded. If the distance traveled by the mouse before falling was less than one quarter of the full length, the immobilization time was recorded as 60 s. In addition, the number of times that the paws slipped was recorded. If the paws clasped the beam while walking, a paw slip number of 2 was recorded for each walk. If the mouse fell within the first quarter of the total distance, a time of 60 was recorded. Data from three trials were averaged for analysis.

### Ledge Test

A simple ledge test was employed to assess balance and coordination of the mice as described previously (Zhu et al., 2016), but with slight modifications. Briefly, each mouse was placed on a ledge (length, 90 cm; width, 0.5 cm; height, 20 cm) and paws placement and forward movement while walking on the ledge were observed. Mice can place their hind paws on the edge, but it is often clasped the edge used their hind paws during walking along the edge under the condition of cerebellar dysfunction.

### Vertical Pole Test

A vertical pole test was employed to assess motor coordination and balance skills. Briefly, a rough plastic pole (height, 50 cm; diameter, 1 cm) was attached vertically to a soft platform, as described previously (Zhu et al., 2016). Then, each mouse was placed facing upward on the top of the pole and the time taken to turn around and climb down the vertical pole was recorded. The maximum time complete the task was 120 s. If the mouse fell from the top of the pole, a time of 120 s was recorded. Each mouse underwent three trials at intervals of at least 10 min. Data from three trials were averaged for analysis.

### Hangwire Test

The hangwire test was conducted to assess balance and grip strength. Briefly, mice were hung upside down on a 12 mm^2^ wire screen suspended 50 cm above a cage, as described previously (Zhu et al., 2016). The time to fall into the cage was recorded. Mice that did not fall within the 60 s trial period were assigned the maximal score of 60 s.

### Traction Test

The traction test was employed to measure grip strength. Briefly, each mouse was allowed to grasp a bar (diameter, 1 mm) with the forepaws and then slowly pulled back by the tail. The maximum tension (in g) before release was recorded and normalized to body weight (Zhu et al., 2016). The mean of three measurements at intervals of 5 min for recovery was determined for analysis.

### Immunohistochemical Analysis

The mice (age, 9–10 months; 2 males and 3 females/ group) were anesthetized with 3.6% chloral hydrate and intracardiac perfusion with phosphate buffered saline (PBS) followed by 4% paraformaldehyde in 0.1 M PBS (PH 7.4). The brain of each mouse was dissected and stored in 4% paraformaldehyde in 0.1 M PBS (PH 7.4) overnight at 4°C and then cryoprotected in 30% sucrose in PBS for 48 h at 4°C. Sagittal sections (thickness, 30 μm) were cut with a cryostat and mounted onto glass slides. Hematoxylin and Eosin (H&E) staining was performed on cryosections. For immunohistochemical analysis, frozen sections were air dried, rinsed with PBS, and then incubated with 0.3% Triton X-100 in PBS for 30 min. Binding of nonspecific antibodies was blocked by treating the sections with blocking buffer (10% bovine serum albumin and 0.3% Triton X-100 in PBS) for 1 h at room temperature. Afterward, the sections were incubated with primary antibodies against TRIM32 (1:200; Abnova, #H00022954-M09; 1:100; Proteintech, #10326-1-AP), calbindin (1:1,000; Swant, #300, and #CB38,); vesicular glutamate transporter 1 (vGlut1) (1:1,000, Synaptic systems, #135302), and vesicular glutamate transporter 2 (vGlut2) (1:1,000, Synaptic systems, #135402). The sections were washed three times with PBS containing 0.3% Triton X-100 for 30 min and then incubated in solutions containing Alexa Fluor^TM^ secondary antibodies (1:1,000, Thermo Fisher Scientific, #A21206, #A21202; #A31570; #A31572). For confocal microscope imaging, the sections were washed three times with PBS and then mounted with Vectashield® antifade mounting medium (Vector Laboratories).

### Golgi Staining

After intracardiac perfusion with 0.9% saline, the cerebellum of each mouse (10 months old, two males and one female/group) was quickly removed and stained using the FD Rapid Golgi Stain^TM^ Kit (FD Neurotechologies, Columbia, MD, USA). The tissue was mounted using Permount^TM^ Mounting Medium (Fisher Scientific) and then dried for 1 week. A total 10 to 15 cerebellar PCs per mouse of a particular genotype were photographed. The size of the soma and dendritic areas of each PC were measured using Image J software as described previously (Kumazawa et al., 2013). Sholl analysis was performed to assess dendritic arborization using Image J software as described previously (Gutierrez and Davies, 2007; Garcia-Segura and Perez-Marquez, 2014). Spine density was determined manually using Image J software. All measurements were obtained from five sections from three mice of each genotype and averaged.

### Quantitative Analysis

For analysis of the size of the cerebellum, quantification was made over 4 HE staining sagittal sections from three mice of each genotype throughout the anterior-posterior extent of the cerebellum on each mouse. Cerebellar outline area was measured in sagittal sections using Image J software (National Institutes of Health, Bethesda, MD, USA). Similarly, for analysis of the thickness of the ML, the lobule VIII was chosen and measured using Image J software.

For quantification of calbindin-positive PCs, five sagittal sections were selected from the same level in each mouse and a 200 μm line was drawn along the entire monolayer of PCs in lobule VIII of each section. Numbers of PCs in 200 μm length Purkinje cells layer were counted. NeuN-positive granule cells in GCL were counted in a 100 × 100 μm^2^ area of lobule V–VII in each sections. To quantify vGlut1-positive PFs-PCs, the mean fluorescence intensity of vGlut1 in a 50 μm^2^ area of lobules V–VII in each section was measured using Image J software. For quantification of vGlut2-positive CFs-PCs, the number of vGlut2 puncta along a 50 μm length of CFs of lobules V–VII in each section was counted using Image J software. All measurements were obtained from five sections from five mice of each genotype and averaged.

### Western Blot

Cerebellum were collected from 6-month-old and 10-month-old *TRIM32*^−^^/–^ mice (two males and three female) and their littermates (three males and two female), and rinsed in PBS. The tissues were lysed in protein extraction solution (iNtRON Biotechnology, Korea) containing a protease inhibitor cocktail (Thermo Fisher) on ice. The supernatant was collected and protein concentrations were determined using the BCA kit (Solarbio, Beijing, China). Then protein samples were subjected to 10% SDS-PAGE and were then transferred to a polyvinylidene difluoride (PVDF) membrane (Millopore). After blocked with 5% bovine serum albumin for 1 h at room temperature, the membranes were incubated with the primary antibodies [antibodies against INPP5A (1:1,000, Invitrogen^TM^, #PA5-45906); antibodies against GAPDH (1:1,000, ZSGB, #TA-08, Beijing, China)] in blacking solution overnight at 4°C. After washed with TBST, the primary antibodies were detected with anti-rabbit or anti-mouse HRP-conjugated secondary antibodies (1:10,000, ZSGB, #ZB-5305; #ZB-5301). The signal was developed using an ECL Prime Chemiluminescent Kit (GE Healthcare). The normalization of the INPP5A was performed relative to GAPDH.

### ELISA Assay

Proteins were gained as above described. IP_3_ ELISA assay were performed according to the manufacture's instruction (MEIMIAN, #MM-0790M2, Jiangsu, China).

### Statistical Analysis

All data are presented as the mean ± SEM. The unpaired two-tailed Student's test (with or without *Bonferroni* correction was performed,) was used for data analysis. All data analyses were conducted using PASW Statistics 20.0 (SPSS, Inc., Chicago, IL, USA). A probability (*p*) value of < 0.05 was considered statistically significant.

## Results

### Balance and Motor Control of TRIM32^–/–^ Mice Were Impaired

**Figure 1:**
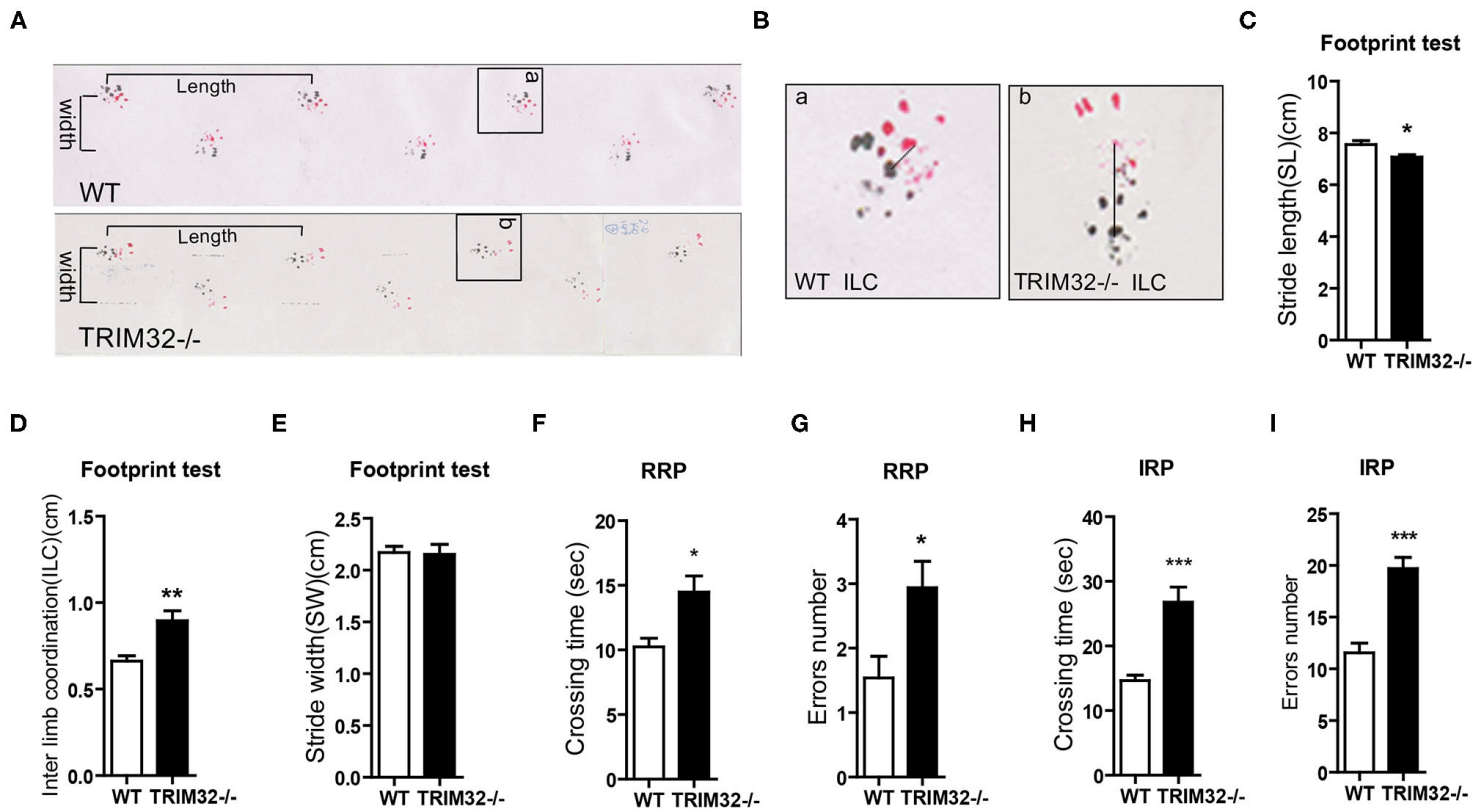
TRIM32 deficiency impairs balance and motor control. **(A)** Representative footprint patterns of adult WT and *TRIM32*^−^^/–^ mice. Stride length and stride width are shown. **(B)** High magnified images of indicated areas in **(C)** show the inter limb coordination of *TRIM32*^−^^/–^ and controls. **(C–E)** Footprint patterns were quantitatively assessed for stride length **(C)**, inter limb coordination **(D)**, and stride width **(E)**. **(F–I)** The ladder rung task was used to evaluate motor coordination. Quantitative analysis of the crossing time and number of limb errors with the regular rung pattern **(F, G)** and the irregular rung pattern **(H, I)**. The performance of *TRIM32*^−^^/–^ mice was poor. Data are presented as the mean ± SEM. Footprint test: n = 11 (*TRIM32*^−^^/–^ mice), = 12 (WT mice); Ladder rung task: n = 13 (*TRIM32*^−^^/–^ mice), = 15 (WT mice). ^*^p < 0.05, ^**^p < 0.01, ^***^p < 0.001.

To determine the role of TRIM32 in balance and motor control, a battery of related behavioral tests were performed using mid-aged (10 months) mice. The results of the footprint test showed that the stride length of *TRIM32*^−^^/–^ mice was reduced (Figures 1A,C) (*t*_(21)_ = 2.742, *p* = 0.012), while interlimb coordination was significantly increased, as compared with the WT mice (Figures 1B,D) (*t*_(21)_ = −3.706, *p* = 0.001), indicating impairment of motor coordination in *TRIM32*^−^^/–^ mice, although no difference when comparing stride width (Figure 1E) (*t*_(21)_ = 0.146, *p* = 0.885). The results of the ladder rung task to assess motor balance and coordination performance (Farr et al., 2006) revealed a greater crossing time (Figures 1F,H) (*t*_(26)_ = −3.110, *p* = 0.004 in regular rung pattern), (*t*_(26)_ = −5.151, *p* < 0.001 in irregular rung pattern) and number of limb errors (Figures 1G,I) (*t*_(26)_ = −2.666, *p* = 0.013 in RRP), (*t*_(26)_ = −5.667, *p* < 0.001 in IRP) for *TRIM32*^−^^/–^ mice with both the regular and irregular rung patterns as compared with the WT mice (Supplementary Video 1). These abnormal behavior performances suggest impaired balance and motor control in *TRIM32*^−^^/–^ mice.

### Motor Coordination and Motor Learning of TRIM32^–/–^ Mice Were Impaired

**Figure 2:**
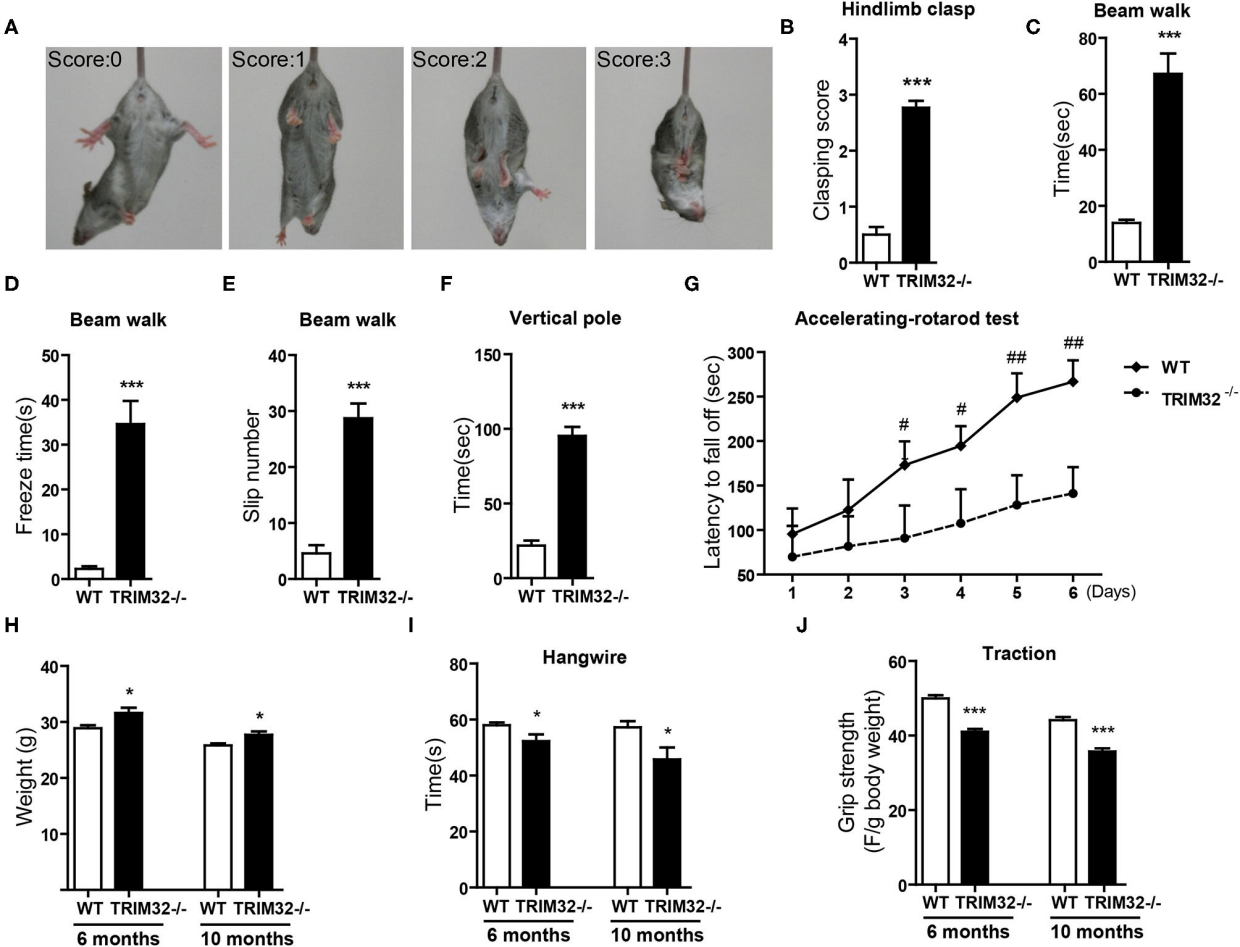
TRIM32 deficiency impairs motor coordination and motor learning. **(A)** Scores assigned to representative postures in the hindlimb clasping test. **(B)** Hind-limb clasping of *TRIM32*^−^^/–^ and WT mice (aged, 10 months) was assessed. **(C,D)** Beam walk test. As compared with WT mice, the performance of *TRIM32*^−^^/–^ mice was poor in regard to the duration to cross the beam **(C)**, freeze time **(D)**, and the number of limb slips **(E)** while crossing the beam. **(F)** For the vertical pole test, the time to turn around and climb down was quantitated. As compared with control mice, the performance of *TRIM32*^−^^/–^ mice was significantly poorer. **(G)** Accelerating -rotarod performance was tested. The time *TRIM32*^−^^/–^ and WT mice remained on the rotarod before falling off was assessed. **(H)** The body of weight of *TRIM32*^−^^/–^ mice and the WT littermate controls were measured at ages of 6 and 10 months. **(I)** For the hangwire test, the latency to fall off by the *TRIM32*^−^^/–^ and WT mice were assessed at the ages of 6 and 10 months. *TRIM32*^−^^/–^ mice displayed impaired motor coordination and muscle deficits. **(J)** Muscular strength of *TRIM32*^−^^/–^ and WT mice at the ages of 6 and 10 months was evaluated using the traction test. *TRIM32*^−^^/–^ mice had weaker forelimb strength. Data are presented as mean ± SEM. n = 13 (*TRIM32*^−^^/–^ mice), = 15 (WT mice). * *p* < 0.05, *** *p* < 0.001, ^#^ *p* < 0.008; ^##^ *p* < 0.001. For the statistical analysis in G, *t*-test with *Bonferroni* correction was performed, *p* < 0.05/6 was considered statistically significant.

The cerebellum is critical to the control of balance, motor coordination, and motor learning. Therefore, cerebellum-related behavioral tests of *TRIM32*^−^^/–^ and WT mice were performed. The hindlimb clasping test was widely applied to assess motor coordination, especially cerebellar ataxia (Chou et al., 2008; Takahashi et al., 2009). The degree of clasping is regarded as a marker of disease severity. Extension reflexes of the hindlimbs and body torsions were normal in WT mice, while clasping of the hindlimb were saw in *TRIM32*^−^^/–^ mice (Figure 2A). Scores of the hindlimb clasping test were significantly higher for *TRIM32*^−^^/–^ mice than WT mice (Figure 2B) (*t*_(26)_ = −12.606, *p* < 0.001). Cerebellum-related motor balance and coordination of *TRIM32*^−^^/–^ mice and WT littermate controls were further assessed. The results of the beam walk test showed that the time spent crossing the beam (Figure 2C) (*t*_(26)_ = −6.195, *p* < 0.001), freeze time (Figure 2D) (*t*_(26)_ = −6.721, *p* < 0.001), and number of paw slips (Figure 2E) (*t*_(26)_ = −8.327, *p* < 0.001) were significantly increase among the *TRIM32*^−^^/–^ mice, as compared to the WT littermates, indicating poor performance by the *TRIM32*^−^^/–^ mice (Supplementary Video 2). In addition, the performance of *TRIM32*^−^^/–^ mice in the ledge test was relatively poor, as it was difficult for the mice to place the hind paws on the edge and the hindlimbs were clasped while moving forward movement along the edge (Supplementary Video 3). Furthermore, *TRIM32*^−^^/–^ mice required more time to turn around and climb down the vertical pole, as compared to the WT mice (Figure 2F) (*t*_(26)_ = −11.030, *p* < 0.001). These results indicate that the motor balance and coordination of *TRIM32*^−^^/–^ mice were impaired.

The cerebellum is also involved in motor learning, which was accessed with the accelerating– rotarod test. The time for the *TRIM32*^−^^/–^ mice to fall off the rod from day 1 to day 6 was lower than that of the WT mice (Figure 2G) (*p* < 0.008). As body weight can affect performance during the rotarod test, the performance of heavy mice performed was poored than that of lighter mice (Brooks and Dunnett, 2009). As compared to WT mice, mean body weight was higher both in mid-aged (10 months) and younger (6 months) *TRIM32*^−^^/–^ mice (Figure 2H) (*t*_(26)_ = −2.704, *p* = 0.012 in 6 months) (*t*_(26)_ = −2.733, *p* = 0.011 in 10 months), in accordance with the results of previous studies (Kudryashova et al., 2009). However, there was no difference in motor performance during the accelerating-rotarod test in younger mice (data not shown). These results indicate that motor learning of mid-aged *TRIM32*^−^^/–^ mice was impaired.

To exclude possible influences of grip strength on motor performance, the hangwire test and traction test were performed to assess grip strength in motor balance and coordination. The results of the hangwire test showed that the time for *TRIM32*^−^^/–^ mice to fall into the cages was slightly reduced (Figure 2I) (*t*_(26__)_ = 2.278, *p* = 0.031 in 6 months) (*t*_(26__)_ = 2.479, *p* = 0.020 in 10 months) and a grip strength was weaker, as determined with the traction test (Figure 2J) (*t*_(26)_ = 7.865, *p* < 0.001 in 6 months) (*t*_(26)_ = 6.877, *p* < 0.001 in 10 months), for both mid-aged and younger (6 months) mice, similar to the results of previous studies (Kudryashova et al., 2009). The results showed that there were significant differences in motor performance between groups.

Together, these results indicate deficiencies in motor coordination and motor learning of the *TRIM32*^−^^/–^ mice, although the possible contribution of reduced grip strength cannot be fully excluded.

### Loss of TRIM32 Reduced the Size of the Cerebellum

It is known that the cerebellum arises from two distinct germinative zones, the rhombic lip and the ventricular zone, during in the embryonic development. As TRIM32 is involved in cell-fate determinant and differentiation of neural precursor cells in the ventricular zone, it was possible that the morphological structure of the cerebellum was altered in *TRIM32*^−^^/–^ mice.

**Figure 3:**
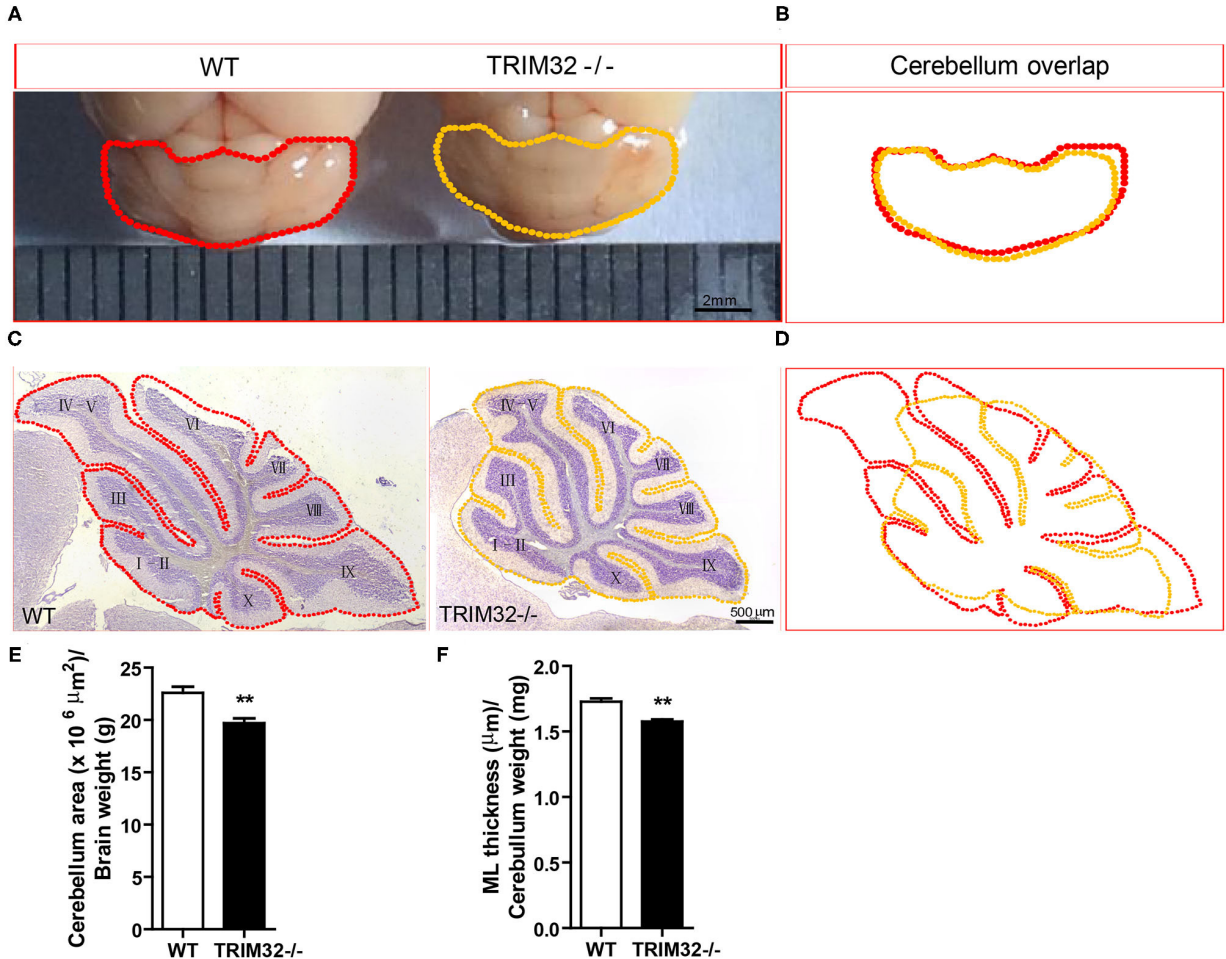
TRIM32 deficiency reduces the size of the mid-aged cerebellum. **(A)** Macroscopic observation of whole cerebella from mid-aged *TRIM32*^−^^/–^ and WT mice. **(B)** Reduced size of the mid-aged *TRIM32*^−^^/–^ cerebellum compared with WT in gross morphology. **(C)** HandE staining sagittal sections of the cerebellum from mid-aged *TRIM32*^−^^/–^ and littermate control mice. **(D)** A smaller cerebellar area in adult *TRIM32*^−^^/–^ compared with control mice in sagittal sections. **(E,F)** Histograms representing the quantification of cerebellar area **(E)** and ML thickness **(F)** of mid-aged *TRIM32*^−^^/–^ and WT mice. Scale bar = 2 mm **(A)**, 500 μm **(C)**. Data were presented as mean ± SEM. n = 5 mice/genotype. ** *p* < 0.01.

To examine this possibility, we first looked at cerebellar gross morphology in macroscopic pictures (Figure 3A) and found no overt morphological alternations between mid-aged *TRIM32*^−^^/–^ and WT littermate cerebella, although there was a mild decrease in the size of *TRIM32*^−^^/–^ cerebella, compared with WT littermates (Figure 3B). Similarly, we did not detect any obvious alteration in the lamination and foliation pattern of mid-aged *TRIM32*^−^^/–^ cerebella (Figure 3C) but again, a slight decrease in cerebellar size (Figure 3D) was detected in *TRIM32*^−^^/–^ cerebellar H&E stained sagittal sections. Within these sections, quantification of cerebellar outline area showed that the cerebellar area of *TRIM32*^−^^/–^ mice was significantly smaller than the cerebellar area of WT mice (Figure 3E) (*t*_(8)_= 3.933, *p* = 0.004). Furthermore, we quantified the thickness of the cerebellar ML and found it was thinner in *TRIM32*^−^^/–^ cerebella compared with the cerebella in WT littermates (Figure 3F) (*t*_(8)_ = 5.214, *p* = 0.001). Together, these results suggest that the loss of TRIM32 reduced the size of the cerebellum, although this loss did not lead to any noticeable morphological structural abnormalities.

### TRIM32 Is Highly Expressed in PCs of the Cerebellum and Deficiency of TRIM32 Results in a Decrease in the Number of PCs

**Figure 4:**
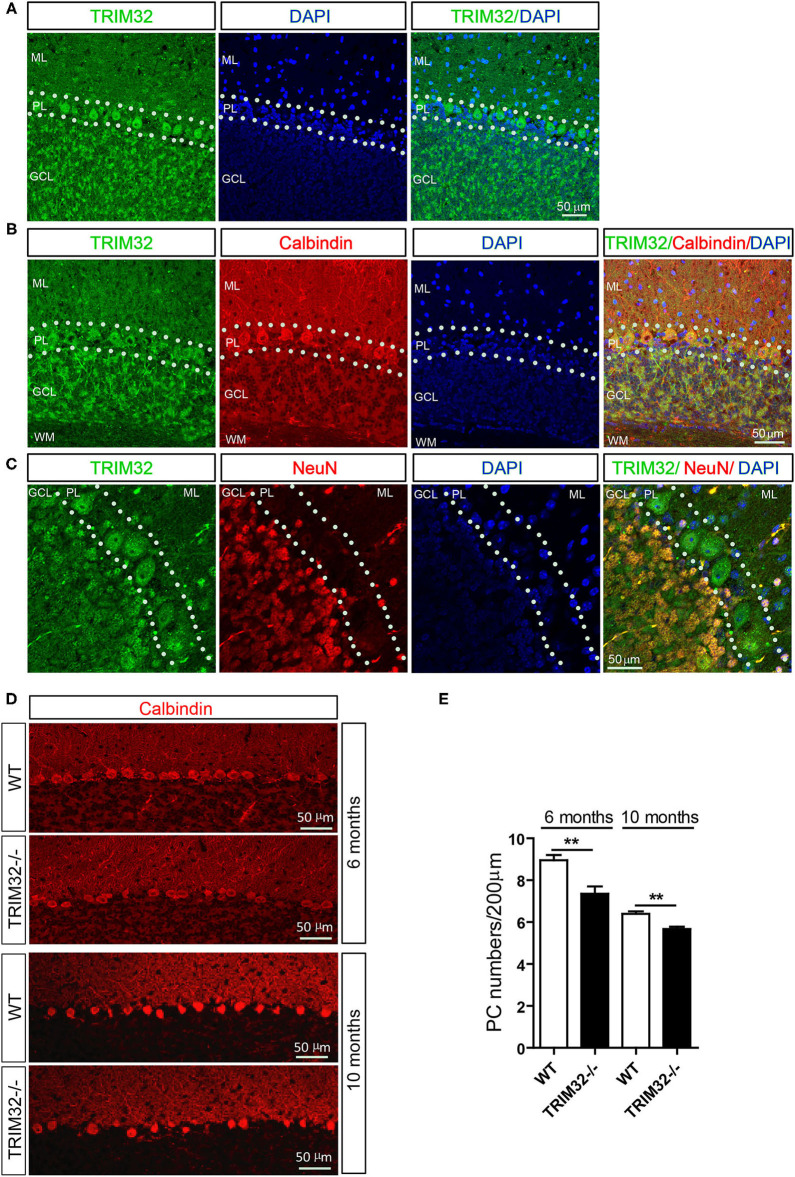
TRIM32 is highly expressed in cerebellar PCs and absence of TRIM32 decreases the number of PCs. **(A)** Cerebellar sagittal sections were stained with specific antibodies against TRIM32. **(B)** Cerebellar sagittal sections were co-stained with antibodies against TRIM32 and calbindin. **(C)** Cerebellar sagittal sections were co-stained with antibodies against TRIM32 and NeuN. **(D)** PCs in the cerebellar sagittal sections were stained with antibodies against calbindin both in 6 months and 10 months old mice. **(E)** The number of PCs was quantified. Scale bar = 50 μm. Data are presented as mean ± SEM. n = 5 mice/genotype. **p < 0.01. ML, molecular layer; PL, Purkinje cells layer; GCL, Granule cells layer; WM, White matter.

To explore the possible influence of TRIM32 in cerebellar function, TRIM32 expression patterns in the cerebellum were examined by immunohistochemical staining. As shown in Figure 4A, TRIM32 was highly expressed in PCs layer (PL) and GCL but no specific immunoreactivity was observed in the *TRIM32*^−^^/–^ cerebellum (Supplementary Figure 2A). Moreover, TRIM32 was co-localized in cerebellar PCs and granule cells that were positively labeled with the PC-specific marker calbindin and granule cells marker NeuN, respectively (Figures 4B,C). Considering that PCs are important neurons for execution of cerebellar function, this result suggests a potential physiological function of TRIM32 in the cerebellum.

The results of our previous study revealed that TRIM32 deficiency leads to reductions in the numbers of GABAergic interneurons in the cortex and hippocampus. PCs are also GABAergic neurons in the cerebellum, but it remains unclear whether the abundance of PCs is also changed in *TRIM32*^−^^/–^ mice. Therefore, quantitative analysis by immunohistochemical staining was performed, which showed that the number of calbindin-positive PCs was decreased in *TRIM32*^−^^/–^ mice, as compared to WT mice (Figures 4D,E) (*t*_(8__)_ = 3.714, *p* = 0.006 in 6 months) (*t*_(8__)_ = 4.536, *p* = 0.002 in 10 months), but no differences was observed in number of NeuN-positive granule cells between in *TRIM32*^−^^/–^ mice and their littermates (Supplementary Figures 2B,C). Thus, these results indicating that deficiency of TRIM32 lead to loss of PCs in the cerebellum and, thereby, impairs motor performance.

### Absence of TRIM32 Leads to Decreased Dendritic Arborization and Spine Density of PCs

**Figure 5:**
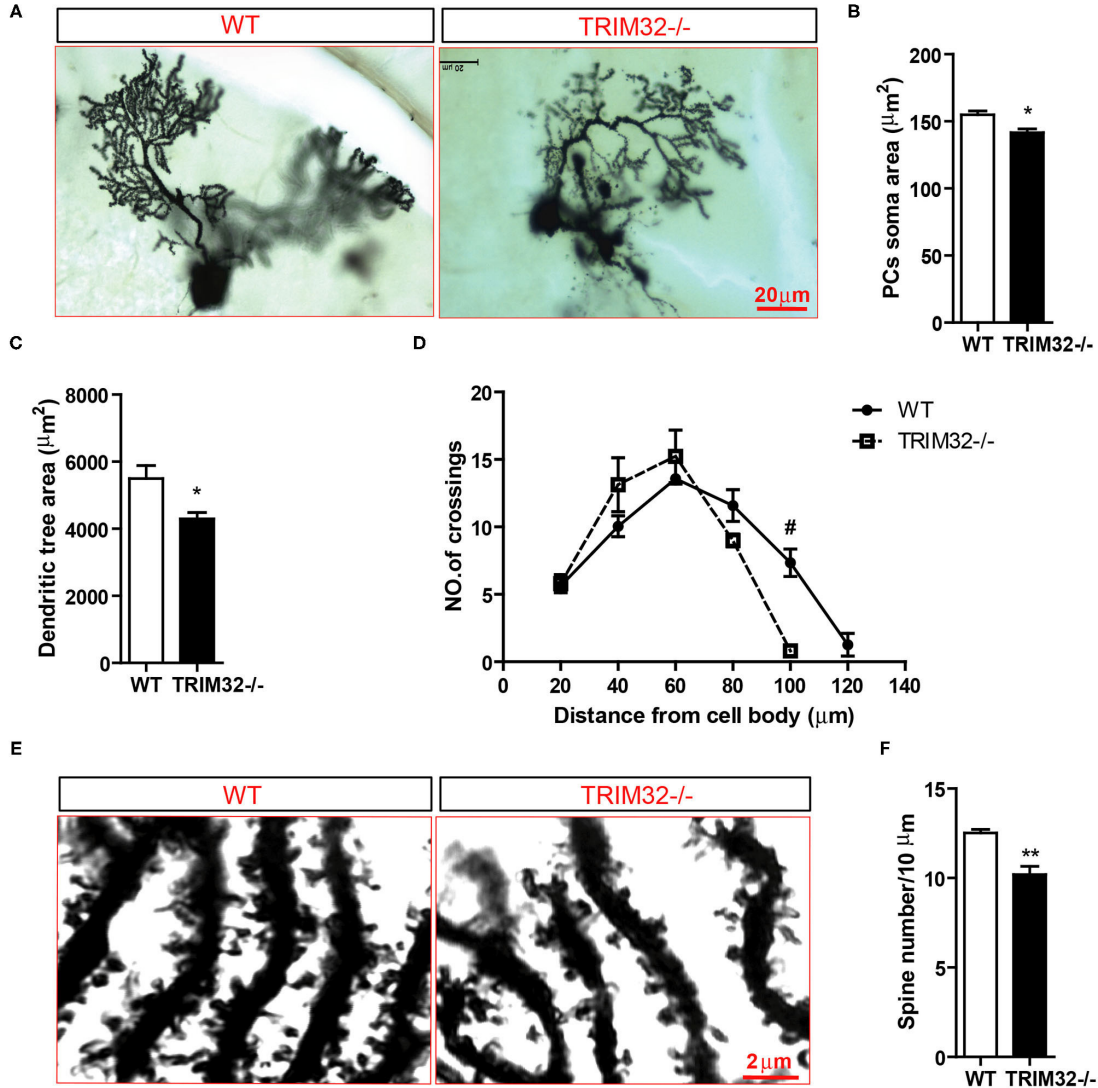
Deficiency of TRIM32 decreases dendritic arborization and spine density of PCs. **(A)** Representative micrographs of stained Golgi of single PCs from *TRIM32*^−^^/–^ and WT mice. **(B,C)** Quantitative analysis of the somata area **(B)** and dendritic tree area **(C)** of PCs. **(D)** Sholl analysis of the numbers of the dendrites at different distances from the cell body. **(E)** High magnified images of distal dendritic branching of PCs. **(F)** Quantitative analysis of the distal dendritic spine density. Scale bar = 20 μm **(A)**, 2 μm **(E)**. Data are presented as mean ± SEM. n = 5 mice/genotype. *p < 0.05; **p < 0.01; ^#^ *p* < 0.008. For the statistical analysis in D, *t*-test with *Bonferroni* correction was performed, *p* < 0.05/6 was considered statistically significant.

In consideration of the importance of the morphology of PCs in the execution of cerebellar function, the morphological characteristic of PCs in *TRIM32*^−^^/–^ and control WT mice were examined. Golgi staining of PCs in sagittal sections of the cerebellum (Figure 5A) revealed significant decreases in the areas occupied by somata (Figure 5B) (*t*_(8)_ = 3.28, *p* = 0.011) and dendritic trees (Figure 5C) (*t*_(8)_ = 2.772, *p* = 0.024) of PCs in the cerebellum between *TRIM32*^−^^/–^ and WT mice. To further investigate possible abnormalities in the morphology of PCs, Sholl analysis of the dendritic arborization of PCs was performed, which found that the distal dendritic arborization of PCs was obviously decreased in *TRIM32*^−^^/–^ mice, as compared with WT mice (Figure 5D) (*t*_(8)_ = 5.325, *p* = 0.003), although there was a slight increase in proximal dendritic arborization. Furthermore, as compared to WT mice, the dendritic spine density of PCs was clearly decreased in *TRIM32*^−^^/–^ mice (Figures 5E,F) (*t*_(8)_ = 4.534, *p* = 0.002). These findings suggest critical roles of TRIM32 in maintenance of dendrite morphology and potential effects on cerebellar function.

### Absence of TRIM32 Leads to Decreased Numbers of PFs-PCs and CFs-PCs

**Figure 6:**
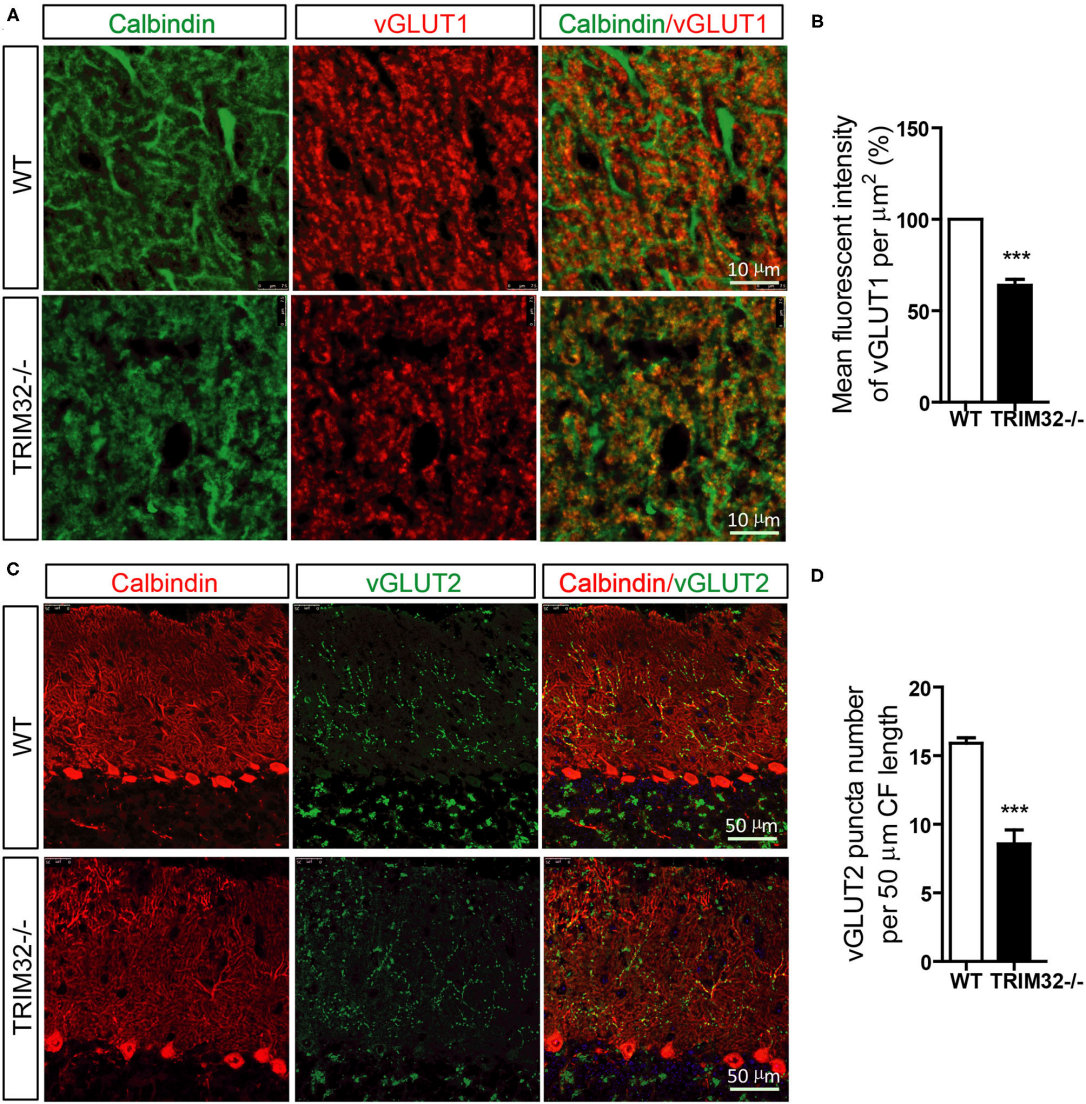
The numbers of PFs-PCs and CFs-PCs were reduced in the *TRIM32*^−^^/–^ cerebellum. **(A)** PFs-PCs and PCs of the cerebellum were stained with antibodies against vGlut1 and calbindin. **(B)** Mean fluorescence intensity of vGlut1 in *TRIM32*^−^^/–^ mice and WT mice. **(C)** CFs-PCs and PCs of the cerebellum were immunostained with antibodies against vGlut2 and calbindin. **(D)** Quantification of vGlut2-positive puncta in *TRIM32*^−^^/–^ mice and WT mice. Scale bar = 10 μm **(A)**, 50 μm **(C)**. Data are presented as mean ± SEM. n = 5 mice/genotype. *** *p* < 0.001.

PFs and CFs are also important for the execution of cerebellar motor functions by forming PF-PCs and CF-PCs to receive information. Therefore, the effect of TRIM32 deficiency on the numbers of PFs-PCs and CFs-PCs was assessed. The results of immunohistochemical staining showed that the mean fluorescence intensity of vGlut1-positive PFs-PCs (Figures 6A,B) (*t*_(8)_ = 10.835, *p* < 0.001) and the number of vGlut2-positive CFs-PCs (Figures 6C,D) (*t*_(8)_ = 6.689, *p* < 0.001) were reduced in *TRIM32*^−^^/–^ mice, as compared to WT mice, indicating that absence of TRIM32 also leads to the loss of PFs-PCs and CFs-PCs in addition to the loss of PCs.

### Deficiency of TRIM32 Decreases INPP5A Level in the Cerebellum

**Figure 7:**
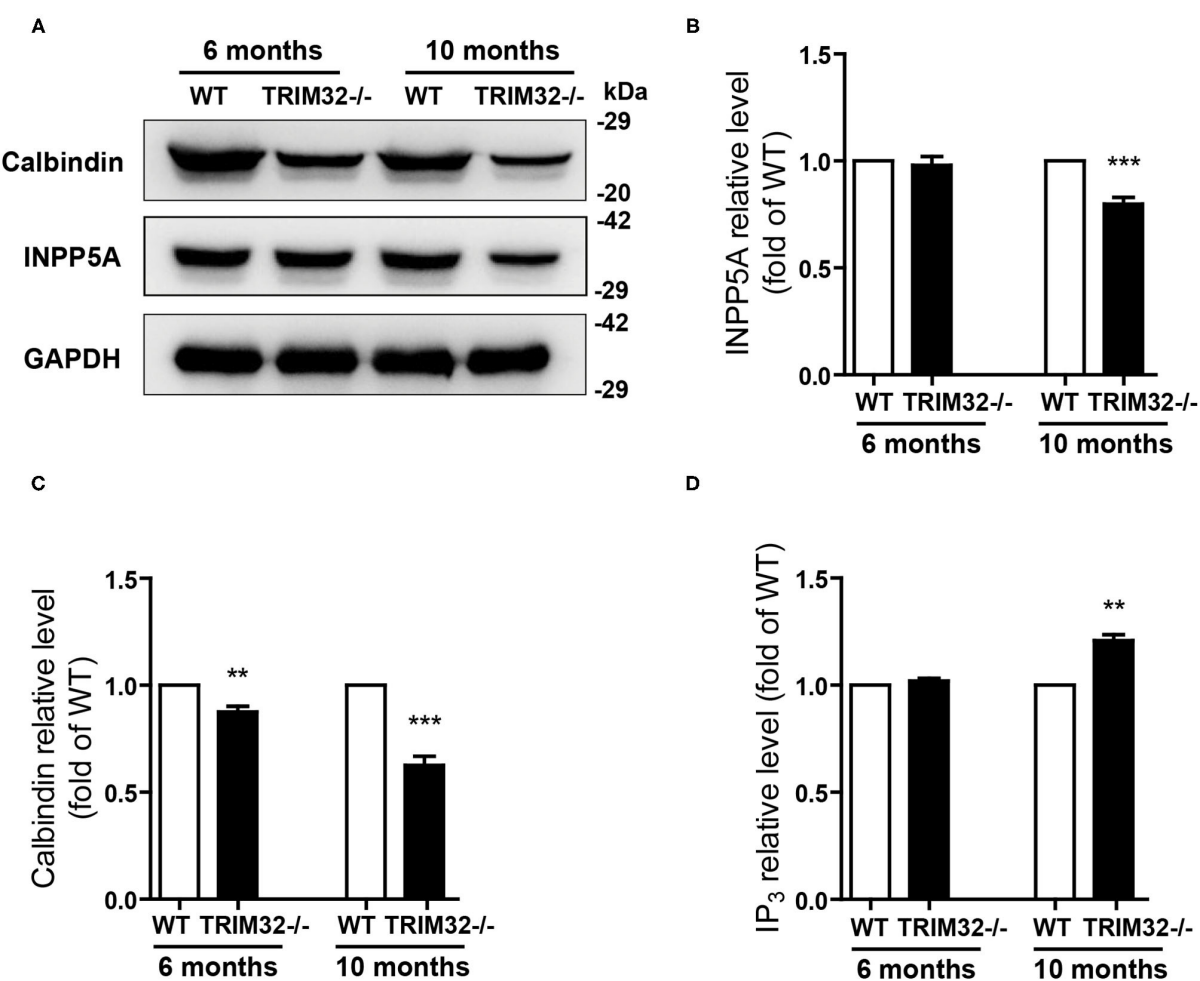
Deficiency of TRIM32 changes levels of INPP5A and IP_3_. **(A)** Western blot analysis of levels of INPP5A and calbindin in *TRIM32*^−^^/–^ mice and their littermates. **(B, C)** Relative levels of INPP5A **(B)** and calbindin **(C)** were quantified. **(D)** ELISA assay analysis of level of IP_3_. Data are presented as mean ± SEM. n = 5 mice/genotype. ** *p* < 0.01, ***p < 0.001.

The above results suggest that cerebellar degeneration may occur in mid-aged *TRIM32*^−^^/–^ mice. Previous studies have shown that INPP5A deletion leads to cerebellar degeneration. To clarify the question, we analyzed the expression level of INPP5A in the cerebellum. As expected, INPP5A level was decreased in the cerebellum in mid-aged *TRIM32*^−^^/–^ mice when compared to their littermates (Figures 7A,B) (*t*_(8)_ = 6.546, *p* < 0.001), but no significant difference were observed in younger *TRIM32*^−^^/–^ mice (6 months), as compared to WT mice (Figures 7A,B) (*t*_(8)_ = 0.509, *p* = 0.624). Meanwhile, we also determinate the relative expression level of calbindin by western blot. Similar to immunohistochemical staining, the level of calbindin was decreased in the cerebellum in *TRIM32*^−^^/–^ mice (Figures 7A,C) (*t*_(8)_ = 4.617, *p* = 0.002 in 6 months) (*t*_(8)_ = 8.861, *p* < 0.001 in 10 months), which indicated that deficiency of TRIM32 causes cerebellar degeneration. IP3 is well known substrates of INPP5A. Disturbance of IP3 signaling has been found in the pathogenesis of SCAs (Liu et al., 2009; Berridge, 2016; Hisatsune and Mikoshiba, 2017). Therefore, we next to analyze the level of IP3 using ELISA assay. Results showed that the IP_3_ level was increased in the cerebellum in mid-aged *TRIM32*^−^^/–^ mice (Figure 7D) (*t*_(8)_ = −4.149, *p* = 0.003), but no difference in younger *TRIM32*^−^^/–^ mice (6 months) (*t*_(8)_ = −1.548, *p* = 0.16). These results indicated that deficiency of TRIM32 caused cerebellar degeneration by decreasing the level of INPP5A in mid-aged mice.

## Discussion

**Figure 8:**
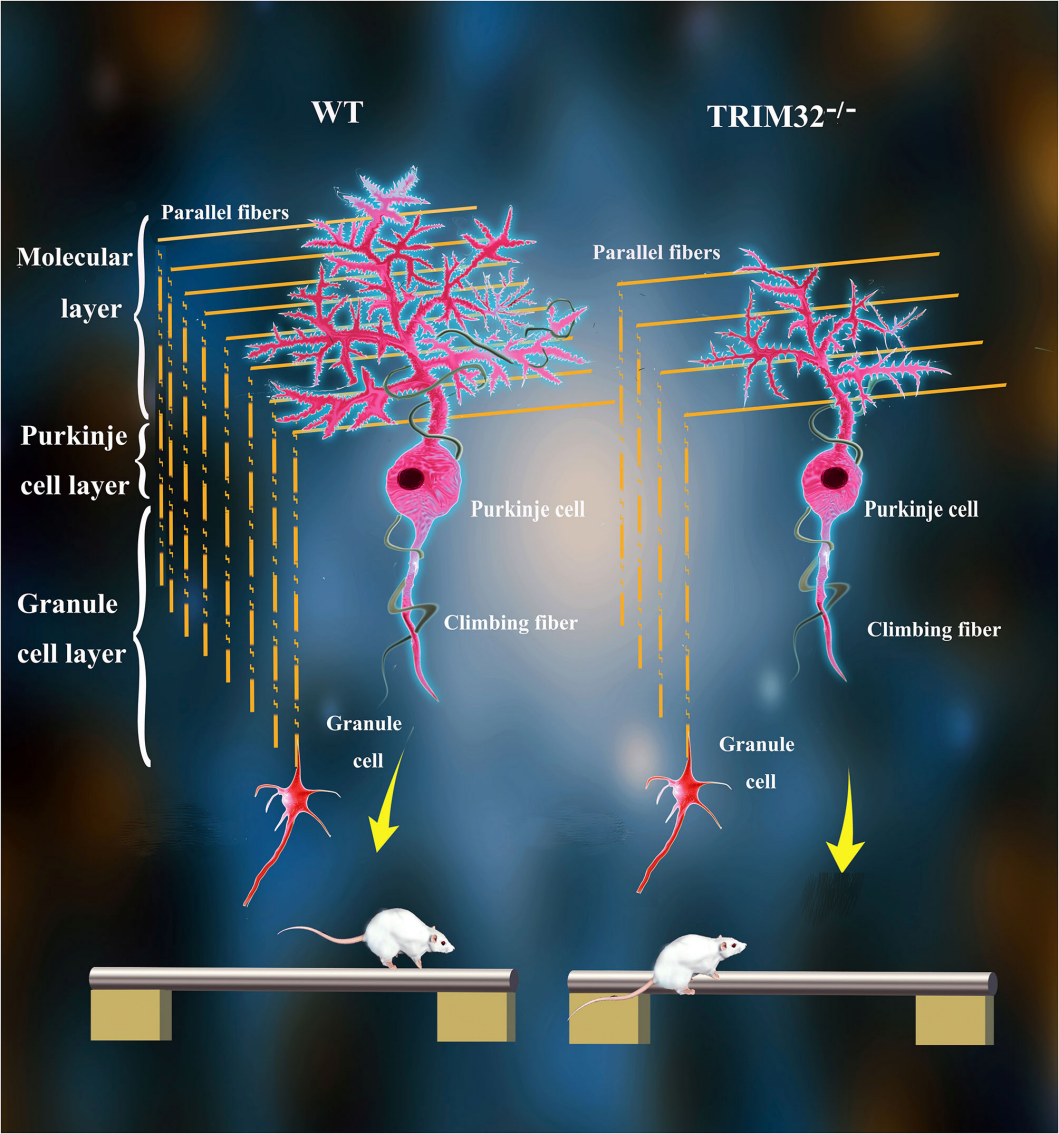
Schematic summary of proposed pathological changes underlying cerebellar motor dysfunction in *TRIM32*^−^^/–^ mice.

The various biological functions of TRIM32 in the CNS have been gradually uncovered over the past 10 years. Remarkably, however, the role of TRIM32 in cerebellar motor function remains unknown. The results of the present study, demonstrated that TRIM32, which is an E3 ubiquitin ligase, is essential for the execution of cerebellar motor function, as well as the survival and morphogenesis of PCs. Moreover, TRIM32 deficiency in mice results in motor dysfunction. Based on the high expression of TRIM32 in PCs, the influence of TRIM32 deficiency on PCs was explored, which found that number of PCs, as well as the dendritic arborization and spine density of PCs, were reduced in the cerebellum of *TRIM32*^−^^/–^ mice. In addition, the loss of TRIM32 causes a decrease in the number of PFs-PCs and CFs-PCs. Furthermore, deficiency of TRIM32 causes INPP5A decreased in the cerebellum, which ultimately leads to cerebellar degeneration (Figure 8).

The cerebellum plays important roles in motor balance and coordination (Mauk et al., 2000; Glickstein et al., 2009). TRIM32-deficient mice exhibited poor performance in different tests of motor balance and coordination skills. Clasping of the hindlimbs is often observed in a number of mouse models of neurodegeneration, including many ataxias (Colomer Gould, 2012) and has been associated with motor neuron degeneration (Takahashi et al., 2009; Guyenet et al., 2010). TRIM32, as an E3 ubiquitin ligase, is closely related to the degeneration of skeletal muscle cells (Cohen et al., 2012; Bernardini et al., 2013). *TRIM32*^−^^/–^ mice exhibited typical hindlimb clasping behavior, which is possibility associated with the degeneration of cerebellar neurons. During the footprint test, stride length was decreased and interlimb coordination was increased, representing alterations to motor function of *TRIM32*^−^^/–^ mice, while there was no change in stride width, probably due to important compensatory mechanisms resulting from axonal projections of the corticospinal tract (Martin, 2005). Impairment of motor coordination was also confirmed by the increased number of limb errors during the ladder rung task, because a misstep or compensatory step by one limb may, in principle, causes a misstep by another limb (Metz and Whishaw, 2002). Poor performance during the accelerating -rotarod test by *TRIM32*^−^^/–^ mice not only indicated impaired motor coordination, but also defective motor learning (Lalonde et al., 1995; McConnell et al., 2009). Cerebellum-related motor balance and the coordination test further confirmed that motor discoordination of *TRIM32*^−^^/–^ mice may be related to cerebellar dysfunction. Motor balance and coordination are based on proper limb placement (Metz and Whishaw, 2002) on the beam or edge. Notably, hindpaw placement and forward movement of the hindlimbs were difficult, as the *TRIM32*^−^^/–^ mice had clasped the beam or edge from the side rather than placing the hind paws on the upper surface. In addition, the results of the vertical pole test revealed impaired motor coordination of *TRIM32*^−^^/–^ mice, which had difficulty reversing direction before climbing down. Moreover, *TRIM32*^−^^/–^ mice also exhibited muscle weakness in both the hangwire test and traction test, which is probably due to limb-girdle muscular dystrophy type 2H caused by TRIM32 deficiency (Kudryashova et al., 2012). Based on these results, it is difficult to determine whether the impairment of motor coordination was due to cerebellar dysfunction, muscle weakness, or both. However, motor function was not impaired in younger (age, 6 months) *TRIM32*^−^^/–^ mice, although grip strength was relatively weakened. Therefore, the poor motor performance displayed by *TRIM32*^−^^/–^ mice may be attributed to cerebellar dysfunction.

The execution of cerebellar function is based on organizational structure (Apps and Garwicz, 2005; Muller Smith et al., 2012) and elaborate neural circuitry (Ito, 2006; Sillitoe and Joyner, 2007; D'Angelo, 2011). During development, the formation of the organizational structure of the cerebellum is dependent on the proper differentiation and migration of various cell types, including PCs, granule cells, BG and interneurons. Although TRIM32 is involved in the regulation of neural progenitor cell proliferation and differentiation (Schwamborn et al., 2009; Hillje et al., 2011; Sato et al., 2011), as well as cell adhesion and migration (Kano et al., 2008; Huang, 2010), we detected no obvious alternations in the lamination and foliation pattern in *TRIM32*^−^^/–^ cerebella. We next focused our attention on cerebellar neural circuit connections. PCs, as the sole output neurons of the cerebellar cortex, play important roles in the cerebellar circuitry by receiving excitatory inputs from PFs and CFs and send information to the vestibular and deep cerebellar nuclei (Chan-Palay et al., 1979; Steuber and Jaeger, 2013). Thus, an adequate quantity of PCs is critical for forming neural circuits in the cerebellum. The results of our previous study indicated that *TRIM32*^−^^/–^ deficiency leads to a reduction in the numbers of GABAergic neurons in the cortex and hippocampus, which likely resulted from suppressed proliferation of GABAergic neuronal precursors during the embryonic period (Zhu et al., 2019). PCs are GABAergic neurons in the cerebellum derived from neuronal precursor cells located in ventricle IV (Sotelo, 2004). Therefore, the reduction in the numbers of PCs in *TRIM32*^−^^/–^ mice in the present study likely resulted from decreased proliferation of precursor PCs during cerebellar development. Meanwhile, appropriate dendritic arborization and dendritic spine density of PCs are also essential to establish neural circuits in the cerebellum. Normal dendritic morphology is critical for the function of PCs, as well as cerebellar function, as confirmed in previous studies (Gao et al., 2011; Verslegers et al., 2014). TRIM32 deficiency causes a reduction in the distal dendritic arborization and dendritic spine density of PCs, which likely result in decreased synaptic connectivity of PFs-PCs, possibly leading to alterations in the number and types of input the PCs receive. On the other hand, decreases in the number of PFs-PCs and CFs-PCs in the absence of TRIM32 may directly result in reduced synaptic connectivity, which ultimately leads to cerebellar motor dysfunction. Meanwhile, the loss of dendritic spines and PFs-PCs and CFs-PCs may also give rise to changes to synaptic plasticity in the cerebellum. Long-term depression at the synapses of PFs-PCs, as the major form of cerebellar synaptic plasticity, is closely associated with cerebellar motor function (Ito, 2001; Jorntell and Hansel, 2006), especially motor learning (Boyden et al., 2004). Thus, it was expected that cerebellar motor learning would be impaired in *TRIM32*^−^^/–^ mice, as observed during the accelerating -rotarod test.

Cerebellar degeneration is also important reason for cerebellar motor dysfunction or ataxia, which is widely found in SCAs (Falcon et al., 2016; Liu et al., 2020). Degeneration of PCs is an obvious hallmark of cerebellar degeneration. Decreased in the number of PCs and in dendritic arborization and axons in PCs are usually found during degeneration of PCs. In the present study, we found the reduced size of the cerebellum, loss of PCs, decreased in dendritic arborization, and spines density of PCs, which suggested that cerebellar degeneration was likely occurred in mid-age *TRIM32*^−^^/–^ mice. INPP5A is a cell type specific protein that is highly abundant in PCs and is involved in cerebellar degeneration (Communi et al., 1996; Liu et al., 2020). In the pathogenesis of SCA17, overexpression of INPP5A ameliorated PCs degeneration and rescued cerebellar ataxia. While knockout of INPP5A in the cerebellum leads to PCs degeneration in mice (Liu et al., 2020). In our study, we found that deficiency of TRIM32 decreased the level of INPP5A in the cerebellum, which is indicated that cerebellar degeneration and possible mechanism responsible for it in mid-aged *TRIM32*^−^^/–^ mice. INPP5A inactivate the second messenger IP_3_ to involve in the transient release of Ca^2+^ from the endoplasmic reticulum (Berridge, 2016). Ca^2+^ signaling in PCs is critical for normal cellular function. Altered IP_3_/Ca^2+^ signaling is closely associated with degeneration of PCs, cerebellar atrophy, and ataxia (Prestori et al., 2019). We provide evidence that increased level of IP_3_ followed by INPP5A decreased in the cerebellum in mid-aged *TRIM32*^−^^/–^ mice. These results indicated that TRIM32 is protected cerebellum from degeneration by maintaining INPP5A level. However, whether altered IP_3_/Ca^2+^ signaling in the cerebellum in *TRIM32*^−^^/–^ mice is still unknown, which is worth investigating this potential correlation in further studies.

The limitation of global deletion of the TRIM32 gene is apparent. In the present study, it was difficult to determine if the impairment of motor coordination was due to cerebellar dysfunction, skeletal muscle weakness, or both. To fully investigate this hypothesis, further investigations of PC-specific conditional TRIM32 knockout mice are necessary.

## Data Availability Statement

The raw data supporting the conclusions of this article will be made available by the authors, without undue reservation.

## Ethics Statement

The animal study was reviewed and approved by the Institutional Animal Care and Use Committee of Sichuan Provincial People's Hospital, University of Electronic Science and Technology of China.

## Author Contributions

R-XX and J-WZ designed the study. J-WZ and W-QJ performed most of the experiments. HZ, Y-FL, and M-MZ contributed to the behavioral testing, Western blot as well as data collection and analysis. Z-TW and B-SW assisted with immunohistochemical analysis, Golgi staining and ELISA analysis. J-WZ wrote and R-XX revised the manuscript. All authors read and approved the final manuscript.

## Conflict of Interest

The authors declare that the research was conducted in the absence of any commercial or financial relationships that could be construed as a potential conflict of interest.

## Publisher's Note

All claims expressed in this article are solely those of the authors and do not necessarily represent those of their affiliated organizations, or those of the publisher, the editors and the reviewers. Any product that may be evaluated in this article, or claim that may be made by its manufacturer, is not guaranteed or endorsed by the publisher.

## Supplementary Material

Supplementary Figure 1 Experimental paradigm of behavioral tests to assess cerebellar motor function.

Supplementary Figure 2 Deficiency of TRIM32 has no effect on granule cells numbers. **(A)** TRIM32 specific antibody was stained in cerebellar sagittal section in *TRIM32*^−^^/–^ mice. **(B)** NeuN-positive granule cells in cerebellar GCL in 10-month-old mice. **(C)** Quantitative analysis of NeuN-expressing granule cells in GCL. Scale bar = 50 μm. Data are presented as mean ± SEM. n = 5 mice/genotype.

Supplementary Video 1 Performance during the ladder rung task. *TRIM32*^−^^/–^ mice (right) walked slowly with frequent limb slips on the bars, as compared to WT mice (left), with both the regular rung pattern (upper) and the irregular rung pattern (lower).

Supplementary Video 2 Performance during the beam walk test. *TRIM32*^−^^/–^ mice (right) walked slowly with frequent limb slips on the beam as compared to WT mice (left). *TRIM32*^−^^/–^ mice had difficulty placing their limbs and clasping the beam when walking forward.

Supplementary Video 3 Performance during the ledge test. *TRIM32*^−^^/–^ mice (right) placed their limbs close to the side walls of the ledge instead of on the ledge surface like WT mice (left) while walking along the ledge. *TRIM32*^−^^/–^ mice exhibited a slower walking pace and a greater number of limb slips than WT mice.

## Abbreviations

ASD: autism spectrum disorder
ADHD: attention deficit hyperactivity disorder
CNS: central nervous system
CFs: Climbing fibers
GL: granule cell layer
PCs: Purkinje cells
PFs: parallel fibers
PBS: phosphate buffered saline
CFs-PCs: synapses between CFs and PCs
PFs-PCs: synapses between the PFs and PCs
TRIM32: tripartite motif-containing protein 32
vGlut1: vesicular glutamate transporter 1
vGlut2: vesicular glutamate transporter 2
WT: wild-type.
