# Supplementary figures and images for: Deficiency of TRIM32 Impairs Motor Function and Purkinje Cells in Mid-Aged Mice
Source: Front Aging Neurosci. 2021 Aug 6;13:697494. doi: 10.3389/fnagi.2021.697494 (PMC8377415; doi:10.3389/fnagi.2021.697494)

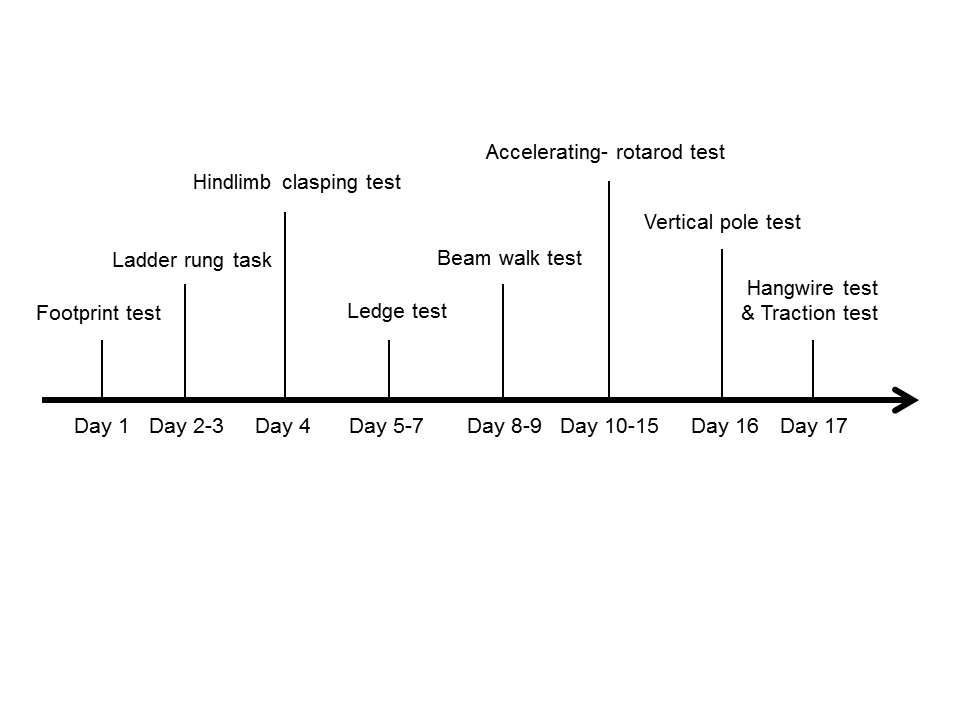

Supplement: Supplementary Figure 1 — Experimental paradigm of behavioral tests to assess cerebellar motor function. [file Image_1.TIF]

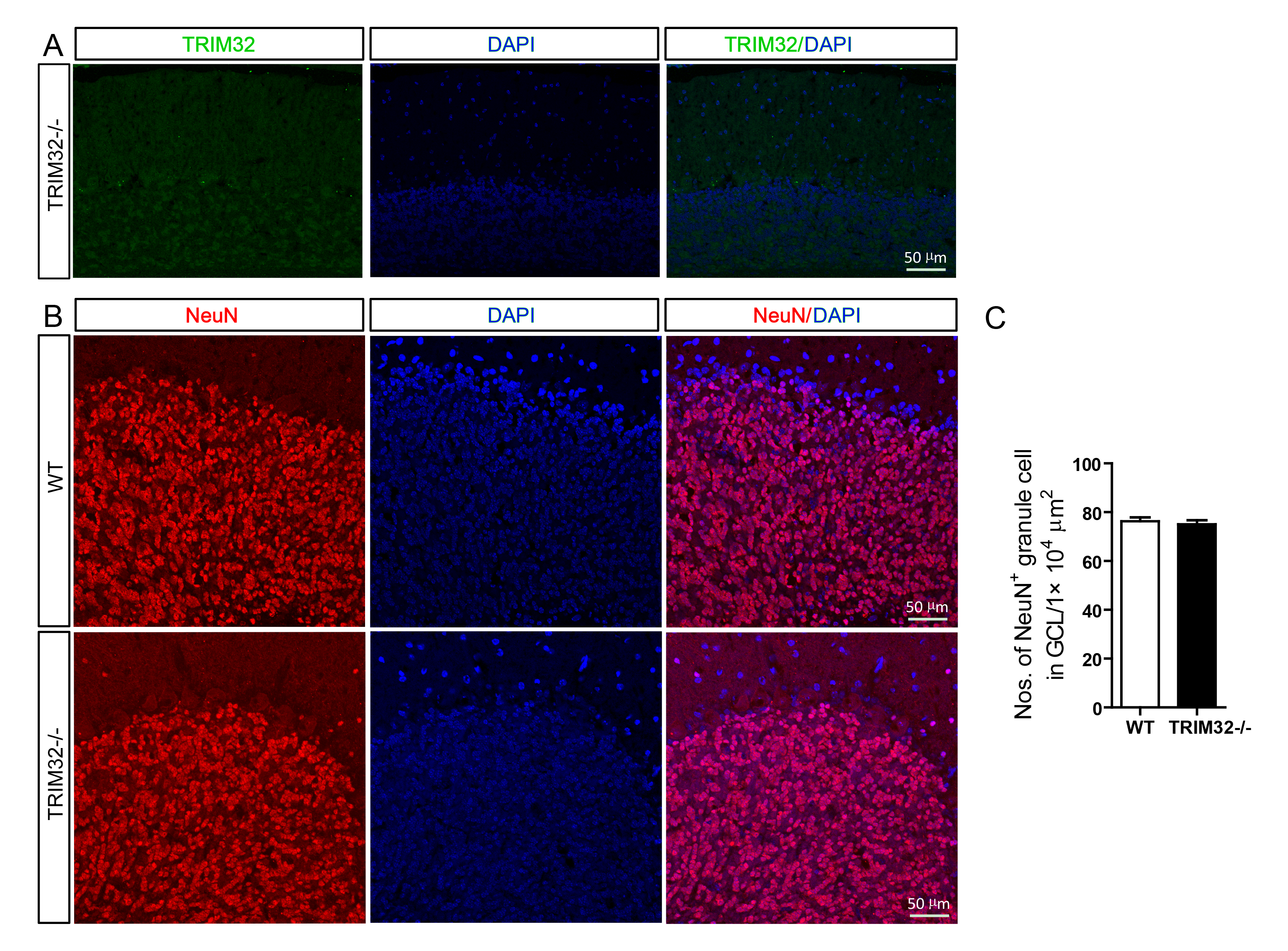

Supplement: Supplementary Figure 2 — Deficiency of TRIM32 has no effect on granule cells numbers. (A) TRIM32 specific antibody was stained in cerebellar sagittal section in TRIM32−/– mice. (B) NeuN-positive granule cells in cerebellar GCL in 10-month-old mice. (C) Quantitative analysis of NeuN-expressing granule cells in GCL. Scale bar = 50 μm. Data are presented as mean ± SEM. n = 5 mice/genotype. [file Image_2.TIF]
